# Evaluation of Dislocation Densities in Various Microstructures of Additively Manufactured Ti6Al4V (Eli) by the Method of X-ray Diffraction

**DOI:** 10.3390/ma13235355

**Published:** 2020-11-26

**Authors:** Amos Muiruri, Maina Maringa, Willie du Preez

**Affiliations:** 1 Department of Mechanical and Mechatronics Engineering, Central University of Technology, Free State, Bloemfontein 9301, South Africa; mmaringa@cut.ac.za; 2 Centre for Rapid Prototyping and Manufacturing, Faculty of Engineering, Built Environment and Information Technology, Central University of Technology, Free State, Bloemfontein 9300, South Africa; wdupreez@cut.ac.za

**Keywords:** direct metal laser sintering, Ti6Al4V(ELI), microstructure, X-ray diffraction, dislocation density

## Abstract

Dislocations play a central role in determining strength and flow properties of metals and alloys. Diffusionless phase transformation of β→α in Ti6Al4V during the Direct Metal Laser Sintering (DMLS) process produces martensitic microstructures with high dislocation densities. However, heat treatment, such as stress relieving and annealing, can be applied to reduce the volume of these dislocations. In the present study, an analysis of the X-ray diffraction (XRD) profiles of the non-heat-treated and heat-treated microstructures of DMLS Ti6Al4V(ELI) was carried out to determine the level of defects in these microstructures. The modified Williamson–Hall and modified Warren–Averbach methods of analysis were used to evaluate the dislocation densities in these microstructures. The results obtained showed a 73% reduction of dislocation density in DMLS Ti6Al4V(ELI) upon stress relieving heat treatment. The density of dislocations further declined in microstructures that were annealed at elevated temperatures, with the microstructures that were heat-treated just below the β→α recording the lowest dislocation densities.

## 1. Introduction

The aerospace and biomedical industries have recently undergone considerable technological advancement in relation to new processes of manufacturing. Additive Manufacturing (AM) is among these new technologies pursued by material scientists and engineers in these industries, as well as in many academic research centres around the globe as a potential replacement of conventional manufacturing technologies such as casting, forging and machining. AM has shown great success and potential and has already been used in the fabrication of functional complex parts with good geometric accuracies, such as novel turbine blades [1] and Leading Edge Aviation Propulsion (LEAP) engine fuel nozzle tips, with a 20-fold reduction in number of parts in an assembly to a single part and a 25% weight saving [2].

Titanium alloys have been used extensively as biomedical implants (orthopaedic prostheses and dental applications), and in the manufacturing of aircraft gas turbine engine components [3]. This is mainly due to their excellent properties of high specific strength (strength normalised by density), good fracture toughness, high fatigue performance, outstanding corrosion resistance and superior biocompatibility [3,4]. Although these alloys have excellent mechanical properties, they are very difficult to machine and require specialised equipment and procedures during casting. This is mainly due to their low thermal conductivity [5] and the acute reactivity of molten titanium with crucibles and mould materials, as well as its affinity for atmospheric gases [6]. The low thermal conductivity of titanium inhibits dissipation of heat within the workpiece itself during machining and thus, the process requires careful selection of machining parameters and large quantities of coolants. Due to the problematic nature of handling molten titanium and its low thermal conductivity, most titanium parts have historically been machined at low speeds [5] from forged titanium ingots. Therefore, the concept of AM has offered a better alternative and has attracted attention in manufacturing of titanium alloys with among them Ti6Al4V, which comprises over 50% of the usage of titanium alloys [3,4,7,8]. In fact, among the limited number of alloys produced via AM, most of the published microstructural details and measurements of mechanical properties have been reported for Ti6Al4V [9].

A review of the literature has revealed that most published work on AM Ti6Al4V aims at producing parts of this alloy with superior mechanical properties that meet all industrial specifications [9]. The AM process is characterised by high energy input in a very short duration of time, which leads to the creation of steep thermal gradients, fast cooling and rapid solidification. This leads to the formation of nonequilibrium microstructures with inferior mechanical properties of deformation. Significant research [10,11,12,13] has, therefore, focused on exploring suitable heat treatment strategies for as-built AM Ti6Al4V parts to achieve the mechanical properties required for industrial applications.

The macroscopic properties of material are largely affected by the microstructure. The microstructure is a very intricate feature with various aspects that influence specific properties in a synergistic manner. These aspects include grain morphology and size, crystallographic texture, internal residual stresses and defects, which are largely dislocations in nature. While strength and ductility are the most important properties for metals and alloys for developing efficient structural components, improving strength often results in degradation of ductility, commonly referred to as strength-ductility trade-off. The strength of materials increases monotonically with the increase in dislocation density; however, this may come at the cost of diminished ductility. Traditionally, strength in metals and alloys has been obtained via cold working and thermo-mechanical processes that introduce very high dislocation densities in materials, and which may also lead to grain refinement due to the formation of high angle grain boundaries [14]. Diffusionless phase transformation of β→α in Ti6Al4V produces highly dislocated martensitic microstructure with high grain refinement [13,14]. Galindo et al. [15] used the model that is commonly used for steels to evaluate the density of dislocations in a martensitic microstructure of AM Ti6Al4V, based on the net strain energy accommodated in a martensitic unit. The authors found the initial dislocation density for this microstructure to be 1.7 × 10^13^ m^−2^. To the knowledge of the authors, there is no other study that has attempted to determine the density of dislocations in the first-order solid-state martensitic microstructure of AM Ti6Al4V (ELI). Information on the extent to which various heat treatment strategies adopted for AM Ti6Al4V (ELI) lower the dislocation density in martensitic microstructures is also lacking.

Dislocation densities in metals and their alloys can be measured through the direct method of Transmission Electron Microscopy (TEM) or indirect methods such as X-ray diffraction (XRD) and neutron diffraction techniques [16]. While the method of TEM can be used for dislocation analysis, it only reveals information about microscopic areas of samples, which raises questions on how representative these areas are of the entire sample volume. In fact, due to the inhomogeneous nature of the first-order solid state martensitic microstructure of Ti6Al4V (ELI) and any other heat treatment state below the martensitic transformation temperature, the dislocation density may vary from place to place. Furthermore, it has been shown in Cong and Murata [17] that for martensitic microstructures with high dislocation densities, applying TEM is difficult due to complicated image contrasts from samples. Under such situations, XRD is a very appropriate technique to investigate the microstructure as it gives information from a macroscopic area in a statistically averaged manner. The method is also appropriate to study the microstructure of a dual-phase Ti6Al4V as the peaks corresponding to constituent phases appear distinctly due to differences of the diffraction vectors (interplanar spacing) [18].

An ideal diffraction pattern consists of narrow, symmetrical, delta function peaks positioned according to a particular {hkil}−plane of a unit cell. The aberrations from the ideal pattern are perceived as peak broadening. The quantification of dislocation density via the XRD method relies on this broadening of diffraction peaks that occurs when atoms in crystal unit cells are displaced from their ideal position due to small crystallite (size broadening) below one micrometre and an abundance of lattice defects (strain broadening) such as dislocations [19]. In order to evaluate dislocation density and the crystallite size, the XRD coherent domain, analytic method, was developed to best fit the diffraction peaks. Its functions consist of those that use the normalised area under a peak Integral Breadth (IB) or Full Width at Half Maximum (FWHM) and those that use the Fourier coefficients of the peaks. The modified Williamson–Hall (MWH) and modified Warren–Averbach (MWA) methods that use IB or FWHM and Fourier coefficients of the peaks, respectively, are the common methods used to determine the dislocation density in polycrystalline materials [20].

### 1.1. Evaluation of Dislocation Density by the Modified Williamson–Hall Method

Williamson and Hall [21] developed a method that simultaneously attributes size (crystallite size) and strain broadening of the peaks to their FWHM. The method is commonly referred to as the Williamson–Hall method and has the formulation:

(1) $$\Delta K≅\frac{0.9}{D}+0.263b\sqrt{\rho }$$

where ΔK  in nm^−1^ is expressed as ΔK=2cosθ(Δθ)/λ and K is the diffraction vector expressed as *K* =2sinθ/λ. In this case, the parameters θ and Δθ stand for the diffraction angle and the FWHM in radians of the XRD peaks, respectively, while λ is the wavelength of the X-rays. The parameters *D, b* and *ρ* represent the crystallite size, Burgers vectors and dislocation density in a material. In this method, the dislocation density in a material can be obtained from the gradient of a linear plot of ΔK obtained from XRD peaks against diffraction vector, *K*, while the crystallite size is obtained from the y-intercept of the plot. Ungar and Borbely [16], showed that in the case of materials that show strong strain anisotropy ΔK is not a linear function of *K*. Strain anisotropy means that the broadening profiles of the peaks show an anisotropic behaviour as a function of the diffraction vector. They modified Equation (1) to account for the influence of strain anisotropy by introducing the average contrast factor of dislocation. This formulation is commonly referred to as the modified Williamson– Hall (MWH) method and is of the form [20]:

(2) $${\left(\Delta K\right)}^{2}≅\;{\left(\frac{0.9}{D}\right)}^{2}+\left(\frac{\pi M^{2}b^{2}}{2}\right)\rho K^{2}\bar{C_{hkl}}$$

The parameter M here is a dimensionless constant depending on the effective outer cut-off radius ($R_{e}$) of dislocations and the dislocation density ($M=R_{e}\sqrt{\rho }$ ). The parameter $\bar{C_{hkl}}$ is the average contrast factor of dislocations, which depends on the relative orientations between the diffraction vectors and the Burger vectors of dislocations, and also depends on the elastic properties of crystals [16]. It has been shown that if all possible slip systems are equally populated with dislocations, the parameter $\bar{C_{hkl}}$ in cases of hcp crystal structures can be expressed by [16,20]:

(3) $$\bar{C_{hkl}}=\;{\bar{C}}_{hk.0}\left(1+\;q_{1}x+\;q_{2}x^{2}\right),\;x=\frac{2}{3}{\left(\frac{l}{Ka}\right)}^{2}$$

where the parameters, $q_{1}$ and $q_{2}$ are curve fitting parameters that depend on the elastic properties of materials, *K* is a diffraction vector, while α and *l* are lattice constants in the basal plane (taken as 0.295 nm for Ti-Hex) and the last index of the (*hkl*) peak reflection, respectively. The parameter $\bar{C_{hk.0}}\;$ is the average contrast factor corresponding to (*hk.0*) reflections, which occur in the planes where the last lattice index, *l*, is zero. To evaluate the parameters *q*_1_ and *q*_2_ therefore the parameter $\bar{C_{hkl}}$, Equations (2) and (3) are normally combined to give a quadratic function of the form:

(4) $$\left({\left(\Delta K\right)}^{2}-\omega \right)/K^{2}≅X\bar{C_{hk.0}}\left(1+\;q_{1}x+\;q_{2}x^{2}\right)$$

where, the parameters *X* and ω are defined as, $X=\pi M^{2}b^{2}\rho /2$ and ω =$\;{\left(\frac{0.9}{D}\right)}^{2}$. As Equation (4) is a quadratic function of x, the values of the parameters, $q_{1}$ and $q_{2}$ in it can be determined from the coefficient of the curve of$\;\left({\left(\Delta K\right)}^{2}-\omega \right)/K^{2}$ against *x*.

### 1.2. Evaluation of Dislocation Density by the Modified Warren–Averbach Method

Warren and Averbach [22] developed a method that expresses the normalised Fourier transforms of the measured broadened peak profiles calculated for equidistant Fourier range *(L)* as the product of the size and the strain coefficient. The expression is normally expressed in a logarithmic scale as:

(5) $$ln\;A\left(L\right)=lnA^{D}\left(L\right)+lnA^{s}\left(L\right)$$

where the functions A(L)*,*$\;A^{D}\left(L\right)$ and $A^{s}\left(L\right)$ are the real, size and strain parts of the Fourier coefficients of the XRD peaks, respectively. When the strain coefficients are expanded and the terms with higher order than *K*_2_ are ignored, this gives rise to the standard formulation commonly known as the Warren–Averbach equation (WA) of the form:

(6) $$lnA_{L}=\;exp\left(-\frac{L}{D}\right)-2\pi^{2}\;L^{2}K^{2}\langle \varepsilon^{2}\rangle$$

where, the parameters L, D and $\varepsilon^{2}$ are the Fourier length, coherent domains of diffraction (crystallite size) and mean square strain, respectively. For materials that show strong strain anisotropic behaviour, Ungar et al. [23] expanded Equation (6) with the theories of Wilkens et al. [24] on the relation between distortion and dislocation density accounting for strain anisotropy, to formulate the modified Warren–Averbach (MWA) equation. Thus, when the strain Fourier coefficients are caused by dislocations and for small values of L, the Fourier transform of the XRD profiles can be approximated as follows [23].

(7) $$lnA_{L}=lnA_{L}{}^{D}-\rho \frac{\pi }{2}L^{2}ln\left(\frac{R_{e}}{L}\right)K^{2}\bar{b^{2}C}$$

In this formulation the product of the square of the average Burgers vectors ($\bar{b^{2}}$) and the average contrast factor $\bar{C_{hkl}}$ is not fitted, but is fixed to a value obtained using the modified Williamson–Hall method (Equation (3)). As seen in Equation (7), $lnA_{L}$ can be considered as a function of$\;K^{2}\bar{b^{2}C}$, thus dislocation density can be determined from a plot of $lnA_{L}$ vs. $K^{2}\bar{b^{2}C}$. The gradient of this plot, *Y*, is the coefficient of the second term on the right side of Equation (7), which can further be expressed as:

(8) $$\frac{Y}{L^{2}}=\rho \frac{\pi }{2}lnR_{e}-\rho \frac{\pi }{2}lnL$$

In the present work, a combination of the MWH and MWA methods was used to determine the dislocation densities of five different microstructures of Ti6Al4V (ELI). One of the microstructures was in the as-built state while the rest were obtained upon heat treatment of this as-built microstructure in different heat treatment cycles ranging from stress-relieving to annealing below and just above the α→β transformation temperature.

## 2. Materials and Methods

### 2.1. Production of Test Specimen for Analysis

The samples for dislocation analysis were fabricated from gas atomised spherical Ti6Al4V (ELI) (ASTM grade 23) alloy powder which was supplied by TLS Technik GmbH (Bitterfeld-Wolfen Germany). The samples were built through the direct metal laser sintering (DMLS) process. The DMLS, a trademark of EOS GmbH (Munich, Germany), is an AM technology that creates components in a layer by layer process from a 3D CAD model by selectively melting and fusing consecutive thin layers with a scanning laser beam. The samples for analysis in the present research were built in an EOSINT M280 machine (EOS GmbH, Munich, Germany) with a standard build layer thickness of 30 µm.

**Figure 1:**
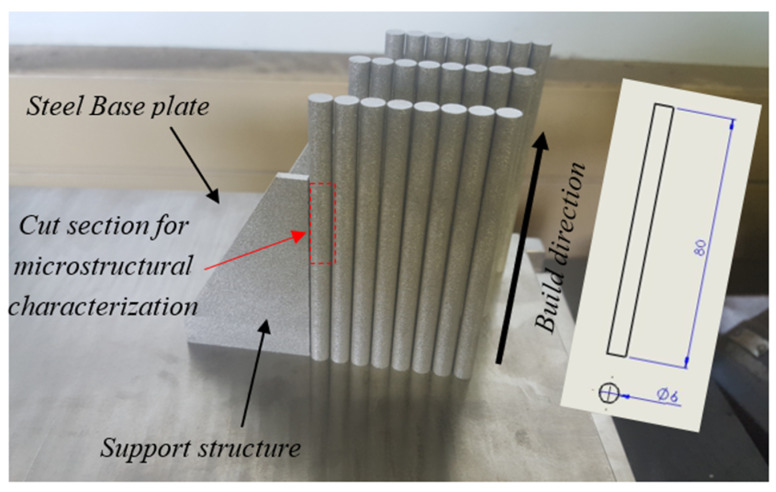
A photograph showing the DMLS-manufactured Ti6Al4V(ELI) test specimens on a steel base plate. The insert schematic drawing shows the dimensions of the specimens in millimeters.

During the fabrication process and after loading the powder onto the build platform, a recoater arm spread the powder evenly across the build platform and then a laser beam selectively scanned the powder. A back and forth raster scanning strategy with rotated strip patterns having a shift angle of 67° after each layer was used in the production of specimens. Cylindrical specimens with a diameter and length of 6 and 80 mm, respectively, were fabricated. A photograph of typical DMLS Ti6Al4V (ELI) samples that were manufactured on the machine build platform in this work is shown in Figure 1.

The manufactured samples for microstructural and dislocation analysis were sub-divided into five groups; designated hereinafter as samples A, B, C, D and E. Among these five groups of the as-built samples, samples B, C, D, and E were further heat-treated in an SS12/24-13MDX Super Series^TM^ vacuum furnace system (T-M Vacuum Products, Inc., Cinnaminson, NJ, USA) with a horizontal vacuum chamber. Samples B were just stress relieved, while samples C, D and E were first stress relieved before subsequently being heat-treated at different high temperatures to allow for microstructural transformation. Stress relieving heat treatment was executed in an atmosphere of argon gas, at a temperature of 650 °C, with a residence time of 3 h, followed by Furnace Cooling (FC) to room temperature in the same atmosphere of argon.

**Figure 2:**
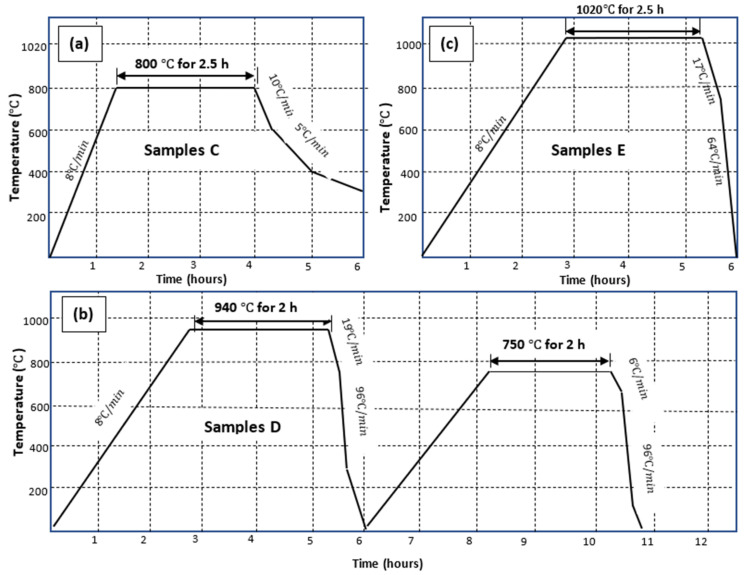
Heat treatment cycles for high temperature annealed DMLS Ti6Al4V (ELI) for (**a**) samples C, (**b**) samples D and (**c**) samples E, following initial stress relieving heat treatment.

The high-temperature heat treatment cycles for samples C, D and E are summarized in Figure 2. The various cooling rates shown in this figure were extracted from the furnace heat treatment profile. Cooling rates affect the growth of α-lathes for temperatures just below and any temperature above the beta transus temperature (980 °C) [25], including formation of martensitic microstructure during very rapid cooling. However, cooling rates have been shown to have minimal effects on the growth of α-grains for temperatures near and below the martensitic transformation (Ms) temperature (780 °C) [26].

Therefore, samples C that were heated slightly above the Ms temperature, were cooled at a furnace cooling rate, which was relatively lower than for the other specimens. This was obtained by switching off the furnace after a set residence time and allowing the chamber to then cool to room temperature. The higher cooling rates for samples D and E were achieved by accelerating the cooling through letting a stream of argon gas into the furnace chamber. The initial slower cooling rates shown in Figure 2 were set to ensure there was sufficient grain growth, while the slightly higher cooling rates thereafter were set to minimize excessive coarsening of the grains formed during cooling, which could have otherwise led to lower values of strength of the final microstructures.

### 2.2. Preparation of Specimens and Methods for Microstructural Analysis

The surfaces of nonheat-treated samples (samples A) and heat-treated samples (samples B, C, D and E) for microstructural analysis were cut from the middle area along the height of the built samples. Small pieces with a height of 12 mm were cut through the diameter from this area (see Figure 1) of the samples and then sectioned into halves, across the diameter with the section planes parallel to the build direction. The cutting was executed using an electrical discharge machine (wire cutting). The cut surfaces were then mounted using a Citopress mounting machine (Struer Cleveland, OH, USA) where the specimens were placed in a mounting cylinder together with Multfast resin. This was followed by chemical and mechanical polishing, before cleaning under tap water and subsequent drying using a stream of compressed air. The surfaces for initial microstructural investigation using a Zeiss Axio Scope A1 optical microscope (Carl-Zeiss Microscopy GMBH, Jena, Germany) were etched using Kroll’s reagent (ES Laboratory, LLC, Glendora, CA, USA), which consisted of 2 mL HF (Hydrofluoric acid), 92 mL distilled water and 6 mL HNO_3_ (Nitric acid).

To study the orientation distribution of grain boundaries in the five groups of DMLS Ti6Al4V (ELI) samples a JOEL JSM-7001 scanning electron microscope (SEM) (JOEL ltd, Akishima, Japan), equipped with an electron backscatter detector (EBDS) for electron backscatter diffraction (EBSD) analysis was used. This system is normally equipped with a low light sensitive camera (CCD) and the HKL channel 5 acquisition and data manipulation software. The samples were mounted and glued onto a holder pre-tilted at an angle of 70° from the horizontal towards the EBSD detector. The EBSD scans on surfaces of the polished samples were conducted on a grid area of 980 µm by 755 µm. The orientation distributions of various microstructures were acquired from EBSD and processed with the HKL channel 5 software (Oxford Instrument HKL, Abingdon, UK). Grain boundary analysis of the EBSD data in the form of (.cft) files was conducted in MTEX, a MATLAB toolbox for analysing and modelling EBSD data.

The qualitative and quantitative analysis to determine the phases present and the dislocation density in five different types of Ti6Al4V (ELI) were performed using an X-ray diffraction (XRD) machine. The XRD analysis was performed on polished specimens that had been used for EBSD analysis, using a Bruker D2 Phaser equipped with a cobalt anode. The specifications of this instrument are shown in Table 1.

The identification of the crystalline phases was done using DIFFRAC.EVA (Version 2, Bruker, Billerica, MA, USA) based on data from the International Centre of Diffraction Data (ICDD) PDF2 database (Release 2016). To minimize the problem of overlapping peaks, thus allowing extraction of clean information from X-ray diffraction patterns, the Rietveld Refinement Technique that is normally implemented in the Bruker AXS TOPAS software (Version 5, Bruker, Billerica, MA, USA) was used to fit the diffraction patterns. Measurements were performed in the angular range 40°(2*θ*) to 120°(2*θ*). Bragg reflections of the {$10\bar{1}0$}, {0002}, {$10\bar{1}1$}, {$10\bar{1}2$}, {$11\bar{2}0$} and {$10\bar{1}3$} planes for the α-phase and the {110} plane for the β-phase of Ti6Al4V (ELI) were measured. The modified Williamson–Hall method and modified Warren–Averbach method were used to evaluate the density of dislocations in samples A, B, C, D and E.

## 3. Results and Discussions

### 3.1. Microstructures of the Nonheat-Treated, and Heat-Treated DMLS Ti6Al4V (ELI)

**Figure 3:**
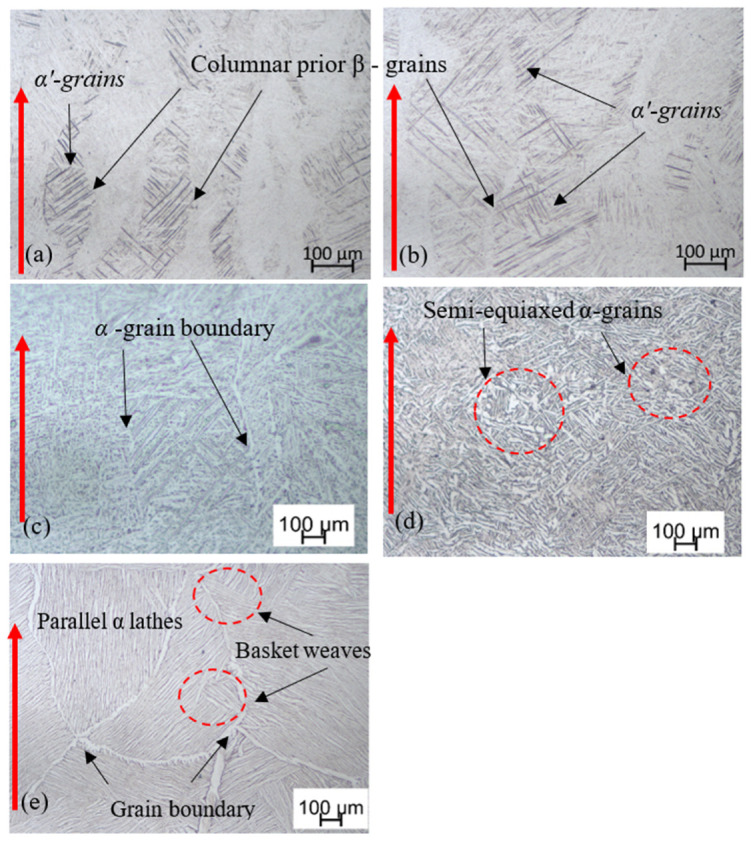
Optical images of (**a**) nonheat-treated samples A and heat-treated samples (**b**) B, (**c**) C, (**d**) D and (**e**) E of DMLS Ti6Al4V (ELI). The red arrows indicate the build direction.

The different microstructures of DMLS Ti6Al4V (ELI) that were obtained before and after various heat treatment cycles are presented in Figure 3. The microstructure of the non–heat-treated material (samples A) is characterized by epitaxial columnar prior-*β*-grains as shown in Figure 3a, with lengths of several millimeters through multiple layers along the build direction. The interior of these columnar *β*-grains contains the acicular *α*′ martensite phase that is needle-like in shape and which arises as a result of high thermal gradients and rapid solidification during the DMLS process. This microstructure remains unchanged after stress relieving heat treatment (samples B), with the columnar prior *β*-grains still filled with α′ acicular grains and remaining elongated along the build direction as seen in Figure 3b.

Samples C were heat-treated slightly above the Ms temperature at 800 °C (see Figure 2), and thus gradual cooling to room temperature transformed the fine acicular *α*′ microstructure to equilibrium mixtures of *α* and *β*-phases, in which the *α*-phase remained present as the needles seen in Figure 3c. The prior *β*-grain boundaries on this micrograph are distinguished in some grains by the precipitation of the *α*-phase at their grain boundaries as shown in the figure, as opposed to the case in the nonheat-treated (Figure 3a) and stress relieved (Figure 3b) microstructures.

Samples D were exposed to duplex annealing (940 °C/FC followed by 750 °C/FC) (see Figure 2), which resulted in a coarse bi-phasic microstructure. Different from samples C (Figure 3c), the α-lathes in this case are larger with an average thickness of about 6 μm as seen in Figure 3d. This microstructure also has some patches of semi-equiaxed grains as seen in the related micrograph.

Samples E, which were annealed above the α→β transformation temperature at 1020 °C (see Figure 2), had the initial columnar prior β-grains transformed into equiaxed and semi-equiaxed prior β-grains as seen in Figure 3e. The microstructure seen in the micrograph in this figure consists of a typical Widmanstätten structure with large α colonies. Additionally, a much thicker continuous grain boundary *α*-phase is observed at the boundaries of the prior *β*-grains.

### 3.2. Misorientation Distribution in Various Microstructures of DMLS Ti6Al4V (ELI)

The grain boundaries between grains in polycrystalline materials are basically regions of disturbed lattice dislocations and are a few atomic diameters in width [26]. Generally, crystallographic orientation changes abruptly in passing from one grain to the next one across grain boundaries. Thus, grain boundaries are conveniently categorised according to the extent of misorientation between adjoining grains. Low Angle Grain Boundaries (LAGBs) are those with a misorientation less than about 15°, while High Angle Grain Boundaries (HAGBs) are those whose misorientation is greater than about 15°.

**Figure 4:**
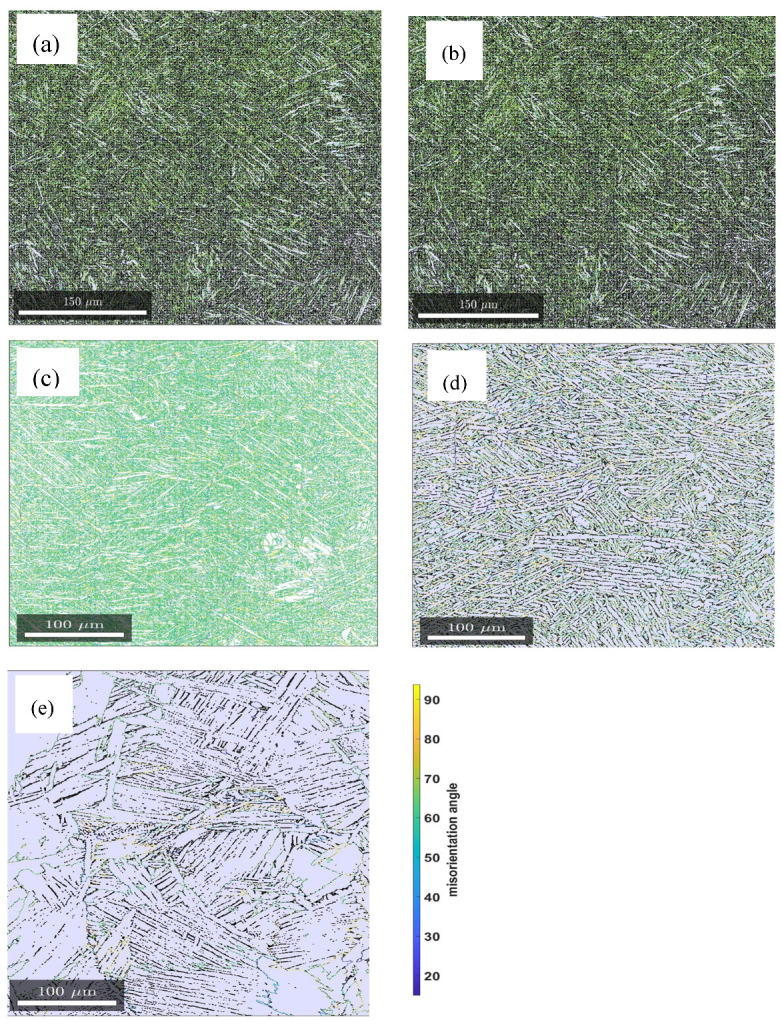
Grain boundary misorientation angles of the samples (**a**) A, (**b**) B, (**c**) C, (**d**) D and (**e**) E.

**Figure 5:**
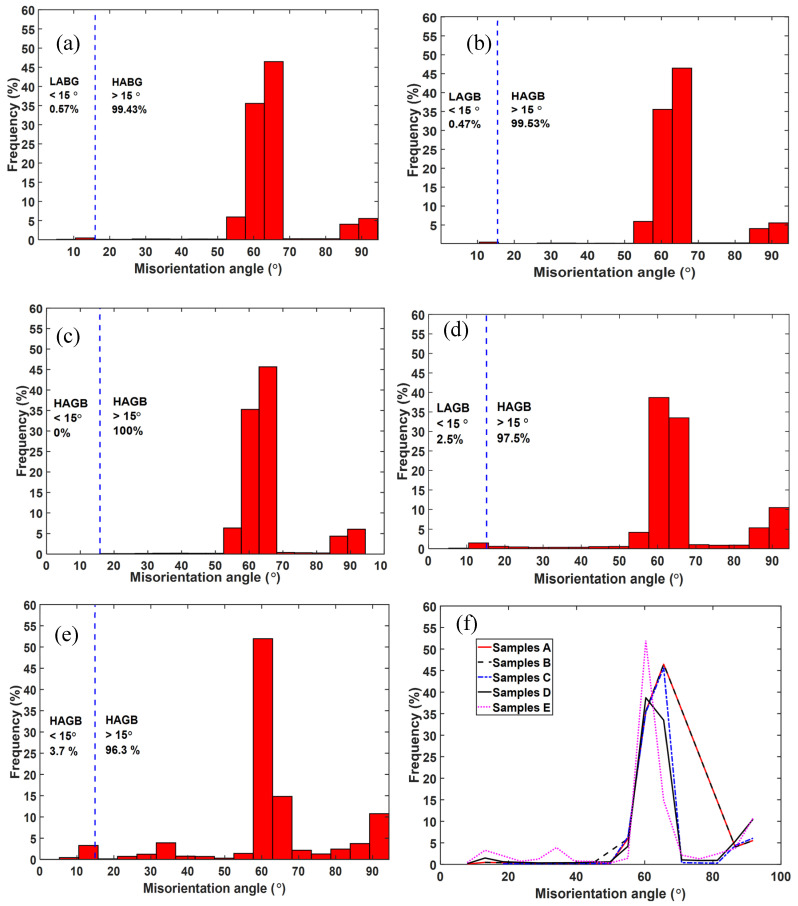
Distribution of grain boundary misorientation angles of the samples (**a**) A, (**b**) B, (**c**) C, (**d**) D, (**e**) E and (**f**) line graph plot for comparison.

In the present research, the EBSD orientation maps were used to quantify the restoration process as a result of heat treatment by measuring the angles of grain boundaries. Figure 4 illustrates the distribution of grain boundary misorientation angles obtained from EBSD scanned surfaces of samples A, B, C, D and E, while Figure 5 provides histograms of the distribution of these misorientation angles.

The percentage fractions of the HABGs accounted for the majority of misorientation angles (>95%) for the five groups of samples with samples C not showing any traces of LAGBs as seen in Figure 4 and Figure 5. While the frequencies of LAGBs for samples A and B are less than 1%, those of samples D and E are 2.5% and 3.7%, respectively. The absence of LAGBs in samples C that were annealed at an intermediate temperature of 800 °C is attributed to annihilation of dislocation arrays and growth of HAGBs at this temperature. Furthermore, the slow cooling rate of this heat treatment ensured minimal or no formation of residual strain, which may manifest as local variation in lattice orientation and therefore generate LAGBs.

The most significant fraction of misorientation is seen in Figure 5 to be at misorientation angles of between 55° and 70° in all cases of the samples scanned. Between these angles the misorientation frequencies of samples A, B and C from misorientation angles 55° to 65° are equal as shown by the coinciding curves in Figure 5f and peak at about 65° for these types of samples. The misorientation frequencies of samples D and E peak at about 60°, showing a decline from the rest. This decline was ascribed to remarkable grain growth that is associated with the two high temperature annealing conditions of samples D and E. This decline would also suggest an ease of slip transfer in samples D and E in comparison to samples A, B and C. It is also noted that the peak frequency of misorientation angles for samples E is 53% compared to a value of 37% for samples D, which is the lowest values for all five types of samples. The high peak frequency in samples E is suggested to be as a result of significant grain growth and thus, existence of relatively few grain boundaries in these samples in comparison to the other samples for an equal EBSD scanned area. In fact, the parallel α-lathes (colonies) seen in Figure 3e would suggest that these grains shared the same crystallographic orientation.

### 3.3. XRD Profile Analysis and Evaluation of Dislocation Densities in Various Microstructures of DMLS Ti6Al4V (ELI)

**Figure 6:**
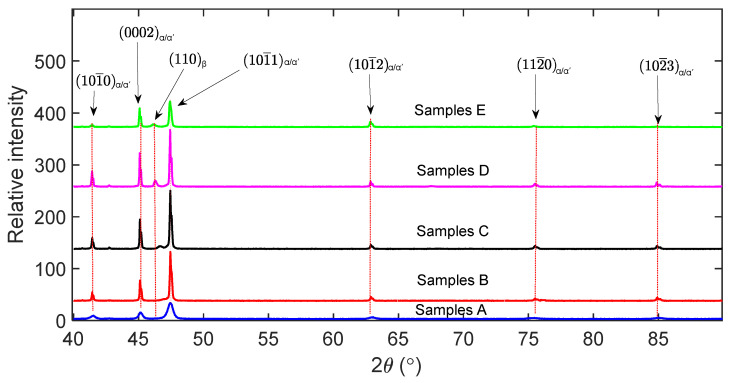
The X-ray diffraction patterns of the as-built (samples A), as-built and stress relieved (samples B), and as-built, stress relieved and annealed samples (samples C, D and E) of DMLS Ti6Al4V (ELI).

The XRD peaks profiles of$\;\left\{10\bar{1}0\right\},\;\;\left\{0002\right\},\;\;\left\{10\bar{1}1\right\},\;\;\left\{10\bar{1}2\right\},\;\;\left\{11\bar{2}0\;\right\}$ and $\left\{10\bar{1}3\right\}\;$ planes for the α-phase and the {110} plane for the β-phase of DMLS Ti6Al4V(ELI) are shown in Figure 6.

The presence of the *β*-phase is noted in samples C, D and E judged from the reflection of the {110}*_β_* plane at angles between 46.10° and 46.69° of the angle 2θ of the XRD profile. From the relative peak intensities of these three types of alloys, the fraction of the *β*-phase in samples C, D and E were determined as 3.6%, 6.4% and 6.6%, respectively.

**Figure 7:**
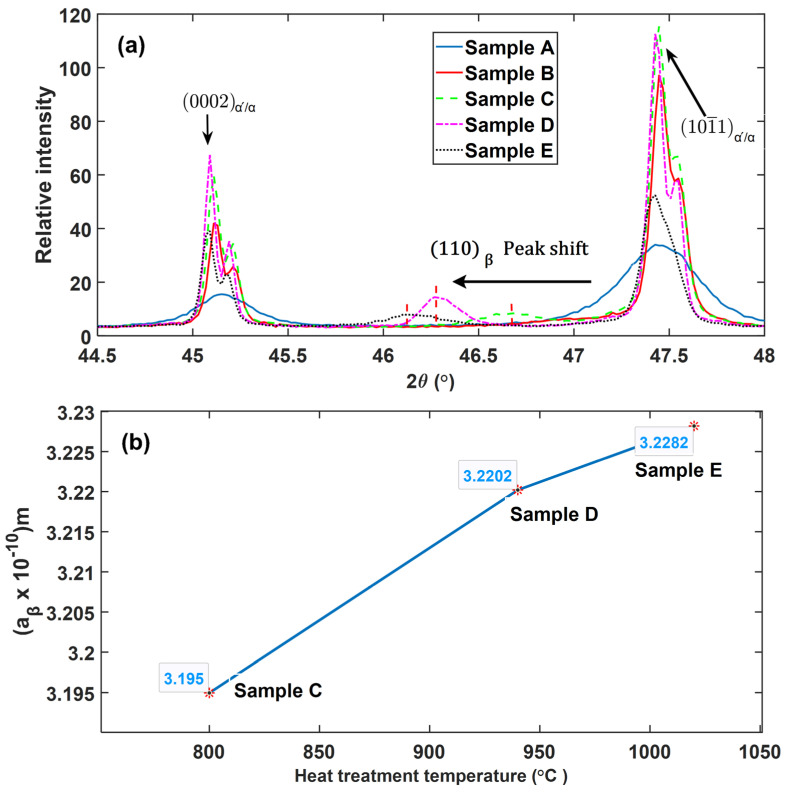
(**a**) The magnification of selected peaks (110)*_β_*, $(\mathrm{0002}{)}_{a՛/a}$ and ($10\bar{1}1$), (**b**) Evolution of the *β*-phase lattice parameter *{a}* with heat treatment temperatures.

The (110) plane for the *β*-phase, and (0002) and ($10\bar{1}1$) planes for the reflections of the α′/α-phase peaks at high magnification are shown in Figure 7a. It is noted in Figure 7a that the {110}—Bragg peak positions of the precipitated *β*-phase in the samples have shifted to a lower angle (2θ) in increasing order of shift angle from samples C, D to E. The shift in the peak position to a lower angle is consistent with an increase in the calculated values of lattice parameter {*a*} of the *β*-phase with increasing heat treatment temperature presented in Figure 7b. The trend observed in Figure 7b agrees well with a recent report from Haubrich et al. [27], which showed the lattice parameter {*a*} of the *β*-phase in Ti6Al4V alloy to increase at elevated annealing temperatures. This increase in magnitude of the lattice parameter is attributed to the decrease in the content of vanadium in the *β*-phase of the Ti6Al4V at high temperatures. For instance, Lasalmonie and Loubradou [28] obtained a 10% vanadium content in the *β*-phase of a Ti6Al4V ingot annealed at 800 °C, while annealing at an elevated temperature of 900 °C decreased the content to 6%. Generally, vanadium has an atomic radius of 0.132 nm while that of titanium is larger at 0.147 nm [29], thus a decrease in the content of the former in the β-phase will result in an increase in the lattice spacing of *β*-titanium.

**Figure 8:**
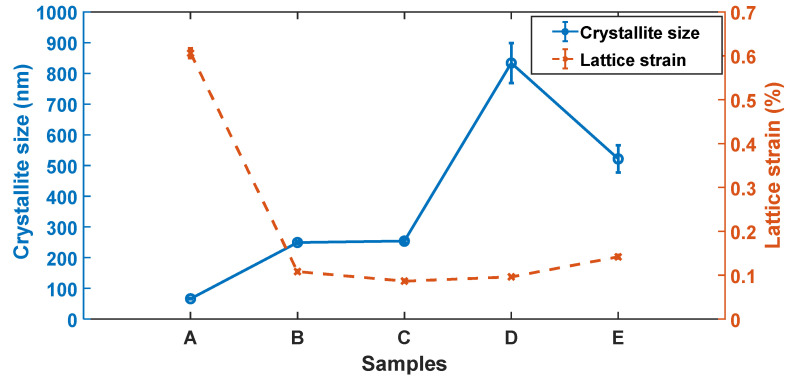
Comparative trend of average crystallite size and lattice strain for various samples of DMLS Ti6Al4V (ELI) as determined by the Rietveld refinement method.

The Bragg peak reflections for the (0002)*_α/α_*, and ($10\bar{1}1$)*_α′/α_* planes in Figure 7a demonstrate a larger integral breath (broadening) of the peaks for samples A compared to the rest of the samples. The broadening of the peaks is normally due to small crystallite size and presence of larger defects in a material [21,22,23]. It should be noted here that the crystallite size are different from grain sizes and the two should not be confused with each other. Crystallite size are sometimes referred to as apparent grain sizes and represent coherent diffraction domains in XRD [30], while actual grains sizes are volumes inside crystalline materials with specific crystal orientations determined via optical or scanning electron microscopes. The average crystallite size and lattice strain of the five groups of the samples that were obtained from Rietveld refinement of XRD data through the Bruker AXS TOPAS software are presented in Figure 8. As seen in Figure 8, the lattice strain and crystallite size are largest and lowest, respectively, in the nonheat-treated samples (samples A). This is consistent with the observation made in Figure 6 and Figure 7 where samples A were observed to have broader peaks in comparison to the rest of the samples. This is expected from the DMLS process, since in this process a small diameter laser interacts with powder feedstock momentarily, thereby producing a molten pool that solidifies rapidly as the laser moves on. This highly localised heat input and short interaction time leads to large thermal gradients and high cooling rates, which result in the formation of fine acicular martensitic microstructure of Ti6Al4V (ELI) with high residual stresses and strains.

Thus, the broader peaks observed in nonheat-treated parts (samples A) are ascribed to: (a) fine martensitic microstructure that gives rise to a small coherent domain, i.e., small numbers of atoms contributing to constructive interference, and (b) assumed large number of defects in the material that create strain and distortion of the lattice. Each defect distorts the lattice in its neighbourhood in a way that leads to a different diffraction profile shape (broadening).

A remarkable decrease in lattice strain and slight increment in crystallite size is noted from samples A to samples B as seen in Figure 8. This is also consistent with the significant decrease in the integral breadth of the (0002)*_α/α′_*, and ($10\bar{1}1$)*_α/α′_* peaks shown in Figure 7a. This is a clear indication of notable reduction of residual stresses in the as-built parts upon exposing them to stress relieving heat treatment. Noteworthy, after annealing at 650 °C for 3 h, there was no observable growth in microstructure with respect to the nonheat-treated condition. However, the observed increase of the scattering domain (crystallite size) could be as result of lattice relaxation that increases scattering volume. It can also be argued that slight grain growth of the martensitic microstructure occurred after stress relieving heat treatment that perhaps could not be detected under an optical microscope.

Samples C recorded the lowest lattice strain, which was attributed to full relief of strain fields associated with lattice distortion expected upon full transformation of strained fine α′ lathes to the thermodynamically more stable α/β lamellae. This assertion is further supported by the fact that in the analysis of misorientation–distribution of grain boundaries (Figure 4 and Figure 5), samples C did not show traces of LAGBs, but were rather characterized by 100% HAGBs. The LAGBs are described in Dieter [31] as containing a simple arrangement of dislocations. Since these dislocations contribute to the distortion of lattices, the increase in lattice strain in order from samples C to E seen in Figure 8 could be attributed to the increasing percentage of LAGBs. The preceding discussion and the histograms shown in Figure 5 clearly underscore the fact of varying dislocation densities between the different microstructures of DMLS Ti6Al4V (ELI) studied here.

The dislocation density in a material is directly proportional to the lattice strain 〈$\;\varepsilon_{l}$〉 and inversely proportional to the crystallite size (*D*), and can be expressed as [32]:

(9) $$\;\rho ≅\;\frac{4\pi \langle \varepsilon_{l}{}^{2}\rangle }{ln\frac{L}{D}Cb^{2}}$$

where the parameters *C* and *b* are the average contrast factor and the Burgers vector of dislocations, respectively. This model clearly shows the correlation between the dislocation density, lattice strain and crystalline size. However, due to long range decay of strain fields created by dislocations, this model cannot be used to calculate dislocation density, since $\langle \varepsilon_{l}{}^{2}\rangle$ has been shown to diverge logarithmically with increasing crystallite size [24]. Thus, the modified Williamson–Hall and the modified Warren–Averbach methods were used to evaluate the dislocation densities in DMLS Ti6Al4V (ELI) samples.

### 3.4. Calculation of Dislocation Densities in DMLS Ti6Al4V (ELI) Microstructures

#### 3.4.1. Analysis of DMLS Ti6Al4V (ELI) XRD Peak Broadening by the Modified Williamson–Hall Method

Diffraction peaks were fitted here using the Gaussian and Cauchy (Lorentzian) functions. The present study was cognizant of the fact that the peaks of the diffractions measured are never exactly described by either one of these functions but instead fall somewhere between the two [22]. The backgrounds of the peak profiles presented in Figure 6 were subtracted and the integral intensity of each reflection normalised first using the equation:

(10) $$\int_{-\infty }^{\infty }I\left(\theta \right)d\theta =1$$

**Figure 9:**
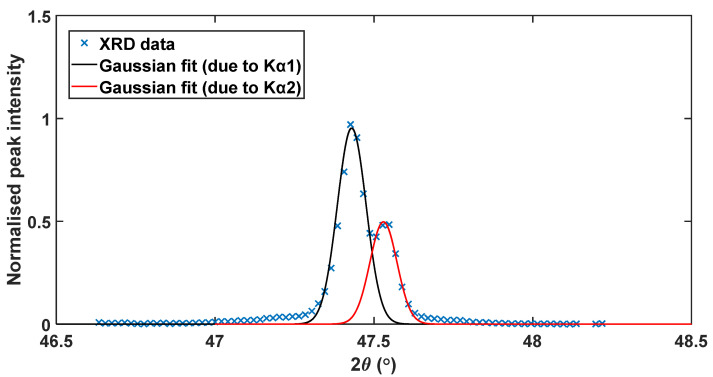
A typical measured XRD data, with the Kα1 and Kα2 deconvoluted peaks using a Gaussian function of ($10\bar{1}1$) reflection.

The normalised peaks were then fitted using Lorentzian and Gaussian functions using *Fityk, an open-source curve-fitting program* that yields peak positions and the FWHM, as well as the integral breadth. It is important to note that the XRD patterns obtained consisted of Kα1 and Kα2 peaks doublet rather than just as single peak as seen in Figure 7a. This is due to the slight difference in wavelength of these two radiations (see Table 1), which according to Braggs law gives rise to a diffraction peak for each wavelength. In the present research only the Co Kα1 radiation were needed and the individual peaks were, therefore, separated into Kα1 and Kα2 using Lorentzian and Gaussian functions. A typical example of the deconvoluted doublet peaks is shown in Figure 9.

**Figure 10:**
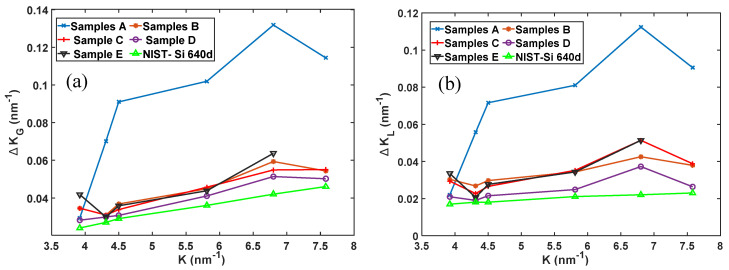
The (**a**) Gaussian and (**b**) Lorentzian FWHM against diffraction vector (*K*) for the five types of the samples with instrumental broadening, referenced against results for a silicon (Si-640d) sample with no instrumental broadening.

Figure 10 shows the trends of the FWHM in nm-1 for the various peaks of the five types of DMLS Ti6Al4V (ELI) microstructures studied here, and that of the National Institute of Standards and Technology (NIST) silicon Si-640d standard sample obtained from the same Bruker D2 Phaser machine. It is apparent from this figure that samples A showed the highest FWHM over most of the diffraction vector positions. As seen in this figure, a remarkable decrease in FWHM was noted upon heat treatment with samples type D recording the lowest values of FWHM over the angular range of the diffraction vectors. To remove instrumental broadening from the measured peak profiles of samples, it was necessary to measure the diffraction pattern of a strain free sample that displays no broadening of its own. A standard reference material, NIST Si-640d was used and its FWHM profiles as a function of diffraction vector (*K*) both as a Gaussian and Lorentzian function are also shown in Figure 10a,b, respectively.

Correction of the effect of instrumental broadening was carried out by subtracting from the FWHM of each peak reflection of the test samples, the values for the Si-640d standard for both the Gaussian and Lorentzian functions. This was done in order to remove instrumental broadening of the peaks to retain the true broadening of the samples using the following equations [33]:

(11) $${\left(\Delta K_{G}^{t}\right)}^{2}={\left(\Delta K_{G}^{m}\right)}^{2}-{\left(\Delta K_{G}^{s}\right)}^{2},\;\Delta K_{L}^{t}=\;\Delta K_{L}^{m}-\;\Delta K_{L}^{s}\;\;$$

In these two equations, the parameters, $\Delta K_{G}^{t}$ and $\Delta K_{L}^{t}$ are the calculated true Gaussian and Lorentzian values of FWHM for the present samples, respectively. The parameters, $\Delta K_{G}^{m}$ and $\Delta K_{G}^{s}$ are the measured values of FWHM for the present samples and the Si-640d reference material in the Gaussian function, respectively, while the parameters, $\Delta K_{L}^{m}$ and $\Delta K_{L}^{s}$ are the measured values of FWHM for the present samples and the Si-640d reference material in the Lorentzian function, respectively.

It is known from the literature [22,23,24] that, crystallite size and strain broadening of the XRD peak profiles are Lorentzian and Gaussian distribution functions, respectively [22]. Thus, the true values of FWHM ($\Delta K_{R}$) due to strain and crystallite size are best expressed by a convolution of Lorentzian and Gaussian functions and in the present case, using the true values of these functions from Equation (11) as follows [33,34]:

(12) $$\Delta K_{R}=\;\frac{1}{2}\left\{1.0692\Delta K_{L}^{t}+\sqrt{0.86639{\left(\Delta K_{L}^{t}\right)}^{2}+4{\left(\Delta K_{G}^{t}\right)}^{2}}\right\}$$

**Figure 11:**
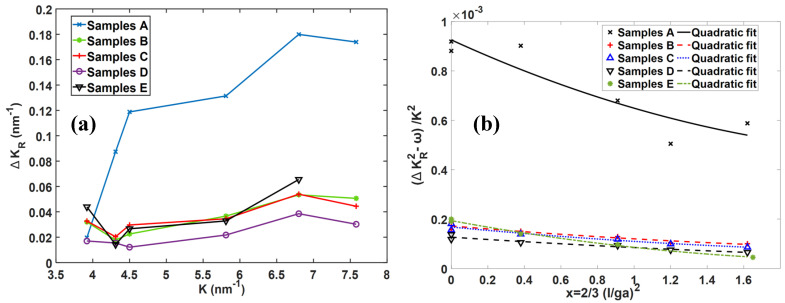
(**a**) The resulting FWHM values of the peaks obtained after convolution of Gaussian and Lorentzian functions in Equation (12) against diffraction vector (*K*) for the five types of DMLS Ti6Al4V (ELI) samples and (**b**) the relationship between $\left(\Delta K_{R}{}^{2}-\omega \right)/K^{2}$ and x for various samples of DMLS Ti6Al4V.

Figure 11 shows the true values of FHWM ($\Delta K_{R}$) obtained from Equation (12) for the five types of DMLS Ti6Al4V samples tested here. Using the values of$\;\Delta K_{R}$ from Figure 11a, analysis was done using Equation (4) that combines the modified Williamson–Hall equation (Equation (2)) and the expression for average contrast factor (Equation (3)). A plot of the function $\left(\Delta K_{R}{}^{2}-\omega \right)/K^{2}$ vs x for samples A to E is shown in Figure 11b. By fitting the quadratic curves of Equation (4) to the experimental values obtained in this work as shown in Figure 11b, values of the parameters $q_{1}{}^{\left(m\right)}$ and $q_{2}{}^{\left(m\right)}$ in this equation that give the best curve fits were obtained and are given in Table 2.

In hexagonal crystal systems such as the ones for Ti6Al4V, there are three different major slip systems that are related to the three glide planes: basal, prismatic and pyramidal [35]. When taking into consideration different slip directions and the character of dislocations (edge and screw) in hcp crystals, there are eleven sub-slip-systems to consider. The values of the parameters *q* and $\bar{C_{hk.0}}$ for each of these eleven sub-slips systems separately, were calculated numerically in work of Dragomir and Ungar [20] and are as shown in Table 3. The fact that the experimental pair values of the parameters, *q* for Ti6Al4V in Table 2 do not match the values of any pair of numerically calculated values in Table 3 suggest that, in practice, more than one sub-slip system is activated during plastic deformation of this material. Dislocations may also be populated in more than one slip system. Therefore the average contrast factor$\bar{\;C_{hkl}}$ (in Equation (3)) for calculating the dislocation density should be evaluated based on the fractions of the three fundamental Burgers vectors in the material shown in Table 3, i.e., **b1** = 1/3<$11\bar{2}0$> (Type <a>) dislocations, **b2** = 1/3<0001> (Type <c>) dislocations and **b3** = 1/3 <$11\bar{2}3$> (Type <c + a>) dislocations. The magnitude of these vectors in case of hcp-titanium are 0.295 nm, 0.468 nm and 0.553 nm for, Type <a> dislocations, Type <c> dislocations and Type <c + a> dislocations, respectively [36,37].

The method of calculating the average Burgers vector and average contrast factor used in a numerous number of researches [16,20,36] for hexagonal crystal structures (hcp) was adopted for use in this research as well. This method is outlined in Appendix A. The values $q_{1}{}^{\left(m\right)}$ and $\;q_{2}{}^{\left(m\right)}$ for the five types of DMLS Ti6Al4V (ELI) samples are presented in Table 2, while the numerical values of$\;\;q_{1}{}^{\left(i\right)}$, $\;q_{2}{}^{\left(i\right)}$ and ${\bar{C_{hk.0}}}^{\left(i\right)}$ for various sub-slip-systems in hcp-titanium are given in Table 3. These two sets of values, from Table 2 and Table 3, were used to determine the unknown fractions of dislocation population, $h_{a}$, $h_{c}$ and $h_{c+a}$, using the three expressions in Equation (A5) in Appendix A.

The values of $h_{a}$, $h_{c}$ and $h_{c+a}$ for the five types of DMLS Ti6Al4V (ELI) samples obtained in this way, were then used to compute the product of the average Burgers vector over the three different slip systems with each Burgers vector and the average contrast,$\;{\bar{b^{2}C_{hk.0}}}^{\left(m\right)}$, using Equation (A3) in Appendix A. The results obtained from these computations are presented in Table 4.

As seen in Table 4, the <a> dislocations dominate the population of dislocations in the five types of samples built here. The <c + a> dislocations are relatively marginal while the absence of the <c> dislocations were noted in all samples. The results presented in Table 4 are in good agreement with the work of Simm [38], where the <a> dislocations were most frequent (>84%) while <c> dislocations were absent. The computed value of ${\bar{b^{2}C_{hk.0}}}^{\left(m\right)}$ for the five different samples of DMLS Ti6Al4V (ELI) shown in Table 4, are very close to one another with a mean value of 0.0249 and standard deviation of 0.00038. These values are also all close to the value obtained in Dragomir and Ungar [20] of 0.0222 nm^2^ for dislocation population fractions of 0.75, 0.2 and 0.05 for <a>, <c> and <c + a> dislocations, respectively. The very small standard deviation of the values shown in Table 4, justified use of the mean value of 0.0249 nm^2^ for ${\bar{b^{2}C_{hk.0}}}^{\left(m\right)}$ in the present work, to determine the dislocation densities in various microstructures of Ti6Al4V (ELI) using the modified Warren–Averbach (MWA) method.

#### 3.4.2. Analysis of DMLS Ti6Al4V (ELI) XRD Peak Broadening Using the Modified Warren–Averbach Method

The real part of the Fourier coefficient ($A_{L}$) of the normalized peaks of XRD profiles can be obtained from the FWHM of Gaussian and Lorentzian fittings as follows [39]:

(13) $$A_{L}=exp-\left\{\left(\frac{1}{2}L^{2}{\left(\Delta K_{G}^{t}\right)}^{2}\right)+L\Delta K_{L}^{t}\right\}$$

**Figure 12:**
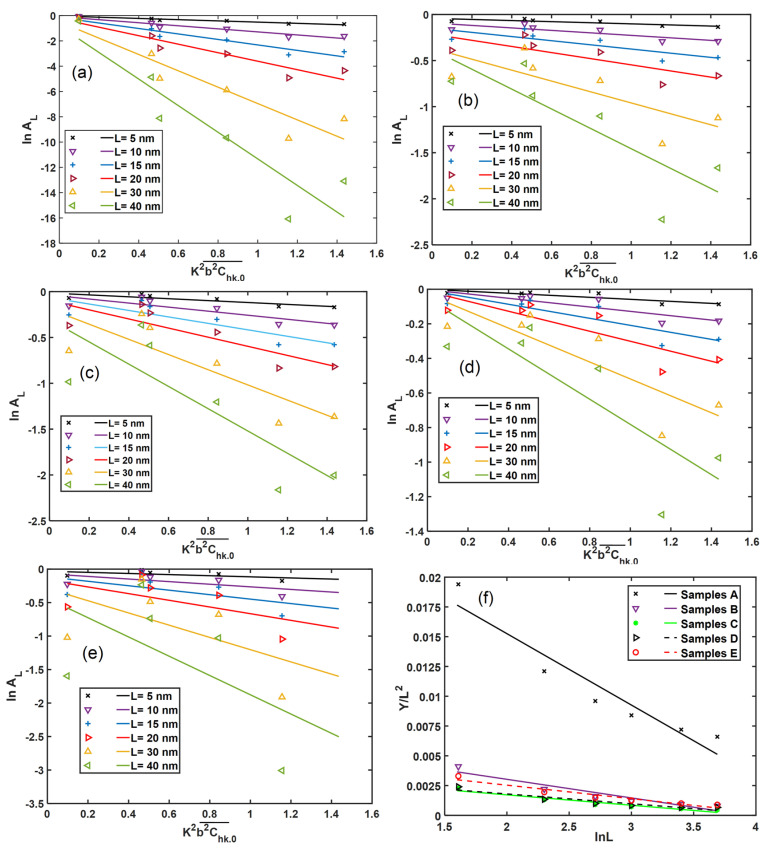
The modified Warren–Averbach plot of DMLS Ti6Al4V (ELI) samples types (**a**) A, (**b**) B, (**c**) C, (**d**) D and (**e**) E according to Equation (7), while (**f**) is a plot according to Equation (8).

Figure 12 shows the modified Warren–Averbach (MWA) plot (Equation (7)) for the five types of DMLS Ti6Al4V (ELI) specimens, obtained by plotting, $lnA_{L}$ vs.$\;K^{2}\bar{b^{2}C}$, for different selected values of Fourier length L. Equation (13) generates Fourier coefficients values that can be fitted with linear curves having gradients that are higher for higher values of Fourier length (L). The gradients of these fitting curves are seen to increase exponentially with increasing L and thus, the visible divergence of the fitted curves at higher values of L. That the plotted data exhibits significant scatter for higher values of L and$\;K^{2}\bar{b^{2}C}$ is not all together surprising as the assumption of the small diffraction vector (K) and Fourier length (L) inherent in the modified Warren–Averbach method do not apply at these higher values [22,39].

**Figure 13:**
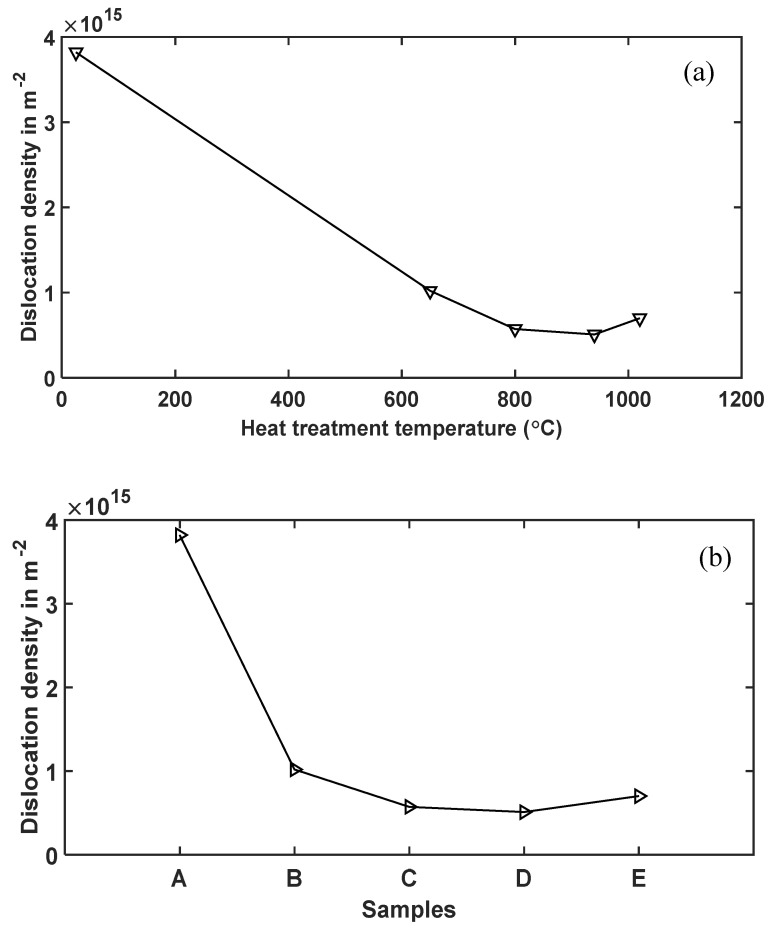
Trend of dislocation density (**a**) with heat treatement temperature and (**b**) in nonheat-treated (samples A) and heat-treated (samples B, C, D and E) microstructures of DMLS Ti6Al4V(ELI).

The slope of the MWA plots in Figure 12a–e give the values of Y, and the ratio, $\frac{Y}{L^{2}}$ against L for various specimens is shown in Figure 12f. The computed dislocation densities of the five different microstructures of DMLS Ti6Al4V (ELI) from Figure 12f are presented in Table 5 and for ease of comparison plotted in Figure 13.

As expected, Figure 13 shows the nonheat-treated (samples type A) microstructure of DMLS Ti6Al4V to have the highest dislocation density among the five different microstructures analyzed in this study. This is anticipated due to distortion of the lattice as a result of the high cooling rates experienced during the DMLS process. The dislocation density is seen in the figure to reduce drastically, by almost 73%, after stress relieving heat treatment (sample type B) conducted at a temperature of 650 °C for 3 h. This reduction was attributed to relaxation of the microstructure and subsequent reduction in residual stresses and lattice strains. The change in dislocation density post stress relieving heat treatment was strictly ascribed to microstructural change. At a heat treatment of 800 °C (samples type C) complete decomposition of the α′-martensite microstructure to an α + β mixture is expected, which in turn lowers the dislocation density as seen in Table 5, with an attendant drop in strength and microhardness of the microstructure. The absence of low angle grain boundaries reported in Figure 4 and Figure 5which are dislocations in nature could also explain the low dislocation density in samples type C. Growth of α-lathes after a heat treatment just below the α→β transformation temperature (samples D) resulted in a further decrease in dislocation density. This type of microstructure recorded the lowest dislocation density (a decrease of about 87% from the one recorded by samples A) as determined by MWA. Interestingly, samples E that were heat-treated above the transformation temperature and had the largest average α-lathe thickness recorded a higher dislocation density than samples C and D and as expected lower than that of samples A and B. This higher dislocation density in samples E than samples C and D is proposed to be as a result of higher percentage of low angle grain boundaries (3.7%) in this microstructure as shown in Figure 4 and Figure 5.

## 4. Conclusions

Misorientation distribution and analysis of XRD profiles for evaluation of dislocation densities in five different microstructures of Ti6Al4V(ELI) produced by DMLS technology were carried out here, with the following conclusions:

The percentage fractions of the HAGBs accounted for the majority of misorientation angles (>95%) for the five groups of samples analysed.

Samples type C did not show any trace of LAGBs (misorientation angles <15°); the frequencies of LAGBS in samples A and B were less than 1%, while in samples D and E they were higher at 2.5% and 3.7%, respectively.

There was no presence of β-phase detected in samples A and B by the XRD method; however, 3.6%, 6.4% and 6.6% of this phase were detected in samples C, D and E, judged from the reflection of {110}-planes.

The {110}—Bragg peak positions of the precipitated β-phase in the samples shifted to a lower angle (2θ) from sample C to D to E in this order, due to increase in the lattice parameters {a} of the β-phase in the Ti6Al4V(ELI) alloy at elevated annealing temperatures.

The lattice strain and crystallite size were highest and lowest, respectively, in the nonheat-treated samples (samples A) and remarkable decrease in lattice strain and increase in crystallite size were noted upon heat treatment.

The nonheat-treated microstructure (samples A) recorded the highest dislocation density of 3.82 × 10^15^ m^−2^ and the highest cut-off dislocation radius of 93.06 nm, both evaluated using the modified Williamson–Hall and modified Warren–Averbach methods.

The dislocation density drastically reduced by almost 73% after stress relieving heat treatment, which is thought to be a result of reduction in residual stresses and strains due to the heat treatment. A further reduction of dislocation density was noted upon subsequent heat treatments, reducing in samples C and D.

There was a noted increase in dislocation density from samples D to E, which is attributed to the higher number of LAGBs in sample E compared to samples C and D.

## Figures and Tables

**Table 1:** Instrument specification: Bruker D2 Phase.

Equipment Part	Parameter		Value
Goniometer Radii	Primary Radius Secondary Radius		70.7 mm 70.7 mm
Detector	2theta angular range		5.638°
Slits	Primary Söller Secondary Söller		2.5° 2.5°
X-ray Source	Co Kα1/Kα2	Operational setting Wavelength	30 KV; 10 mA 0.1788/0.1792 nm

**Table 2:** The measured value of parameters *q*_1_^(*m*)^ and *q*_2_^(*m*)^.

Alloy	$q_{1}{}^{\left(m\right)}$	$q_{2}{}^{\left(m\right)}$
Samples type A	−0.34	0.04
Samples type B	−0.37	0.05
Samples type C	−0.37	0.06
Samples type D	−0.38	0.05
Samples type E	−0.50	0.04

**Table 3:** Numerical calculated values of$\;\bar{\;C_{hkl}}$, $q_{1}$ and $q_{2}$ for the most common slip systems in titanium [20].

Slip System	Slip Plane	Burger Vector	${\bar{C}}_{hk.0}$	$q_{1}$	$q_{2}$
Basal <a>	(0002)	$\left[11\bar{2}0\right]$	0.20227	−0.101142	−0.102623
Prismatic <a>	$\left(1\bar{1}00\right)$	$\left[11\bar{2}0\right]$	0.35387	−1.19272	0.355623
Prismatic <c>	$\left(1\bar{1}00\right)$	[0001]	0.04853	3.61619	1.226411
Prismatic <c + a>	$\left(1\bar{1}00\right)$	$\left[11\bar{2}3\right]$	0.10247	2.01717	−0.616631
Pyramidal1<a>	$\left(10\bar{1}1\right)$	$\left[11\bar{2}0\right]$	0.3118	−0.89401	0.183311
Pyramidal2 <c + a>	$\left(11\bar{2}2\right)$	$\left[11\bar{2}3\right]$	0.09227	1.29905	0.397247
Pyramidal3 <c + a>	$\left(11\bar{2}1\right)$	$\left[11\bar{2}3\right]$	0.09813	1.89412	−0.365739
Pyramidal4 <c + a>	$\left(10\bar{1}0\right)$	$\left[11\bar{2}3\right]$	0.09323	1.52702	0.146150
Screw <a>	Multiple	$\left[11\bar{2}0\right]$	0.1444	0.59492	−0.710368
Screw <c + a>	Multiple	$\left[11\bar{2}3\right]$	0.41873	1.25714	−0.94015
Screw <c>	Multiple	[0001]	3.61 × 10^−6^	165366	−98611

**Table 4:** The fractions of Burgers vector population and the ${\bar{b^{2}C_{hk.0}}}^{\left(m\right)}$ values for the five different samples of DMLS Ti6Al4V in the present work, based on the *q*-values in Table 2 and Table 3, and based on the modified Williamson–Hall method.

Samples	Burgers Vector Population (Fraction)			Average Contrast
	$<a>\;(h_{a})$	$<c>\;(h_{c})$	$<c+a>\;(h_{c+a})$	${\bar{b^{2}C_{hk.0}}}^{\left(m\right)}$
A	0.8800	0	0.1200	0.0253 nm^2^
B	0.9088	0	0.0912	0.0245 nm^2^
C	0.9077	0	0.0923	0.0245 nm^2^
D	0.8819	0	0.1181	0.0252 nm^2^
E	0.8925	0	0.1075	0.0249 nm^2^
				Mean 0.0249 ± 0.00038

**Table 5:** The density and effective outer cut-off dislocation radius resulting from the MWA method.

Sample Type	Dislocation Density (m^−2^)	Outer Cut-Off Dislocation Radius (Re) (nm)
A	3.82 × 10^15^	93.06
B	1.02 × 10^15^	70.02
C	5.73 × 10^14^	46.12
D	5.09 × 10^14^	48.81
E	7.00 × 10^14^	78.54

## Appendix A

The method of calculating the average Burgers vector and average contrast factor is outlined in Ungar and Borbely [16], Dragomir and Ungar [20] and Takashi et al. [36] for hexagonal crystal structures. The measured contrast factor of a material as a product of the average magnitude of the Burgers vector can be expressed as:

(A1) $${\bar{C_{hk.l}b^{2}}}^{\left(m\right)}=\;\overset{N}{\underset{i=1}{\sum }}f_{i}{\bar{C}}^{\left(i\right)}b_{i}{}^{2}$$

where the parameter *N* stands for the number of activated sub-slip-systems (*N* = 11 as shown in Table 5) and $f_{i}$ the fractions of the particular sub-systems defined by Burgers vector $\left(\overset{11}{\underset{i=1}{\sum }}f_{1}=1\;\mathrm{and}\;f_{i}\geq 0\right)$. Equation (A1) is expanded to give the following expression:

(A2) $${\bar{C_{hk.l}b^{2}}}^{\left(m\right)}=\;b_{1}{}^{2}\overset{N<a>}{\underset{i=1}{\sum }}f_{i}\bar{C^{\left(i\right)}}\;b_{1}{}^{2}+b_{2}{}^{2}\overset{N<c>}{\underset{j=1}{\sum }}f_{i}\bar{C^{\left(j\right)}}\;+\;b_{2}{}^{2}\overset{N<c+a>}{\underset{j=k}{\sum }}f_{i}\bar{C^{\left(k\right)}}\;$$

where the parameters N<a>, N<c> and N<c+a> are the number of sub-slip systems with Burgers vectors types <a>, <b> and <c+a>, respectively. With the assumption of any Burgers vector type to be randomly distributed in different sub-slip systems, Equation (A2) is written as:

(A3) $${\bar{b^{2}C_{hk.0}}}^{\left(m\right)}=\sum_{i=1}^{3}h_{i}{\bar{C}}^{\left(i\right)}b_{i}{}^{2}$$

where the parameters $h_{i}$ represent the fractions of the dislocation population,$\;h_{a}$, $h_{c}$ and $h_{c+a}$, in the material with the same Burgers vectors, i.e., **b_1_,** **b_2_** and **b_3_** and ${\bar{C}}^{\left(i\right)}$ are averaged over the sub-slips-systems, each corresponding to the same Burgers vector type. The average contrast factor (Equation (3)) is substituted into Equation (A3) to give rise to:

(A4) $$\overset{3}{\underset{i=1}{\sum }}h_{i}{\bar{C_{hk.0}}}^{\left(m\right)}b_{i}{}^{2}\left(1+\;q_{1}{}^{\left(m\right)}x+\;q_{2}{}^{\left(m\right)}x^{2}\right)=\;\overset{3}{\underset{i=1}{\sum }}h_{i}{\bar{C_{hk.0}}}^{\left(i\right)}b_{i}{}^{2}\left(1+\;q_{1}{}^{\left(i\right)}x+\;q_{2}{}^{\left(i\right)}x^{2}\right)$$

The measured values of$\;q_{1}{}^{\left(m\right)}$ and$\;q_{2}{}^{\left(m\right)}$ are written in terms of numerically calculated values of$\;q_{1}{}^{\left(i\right)}$, $\;q_{2}{}^{\left(i\right)}$ and average contrast factor (${\bar{C}}^{\left(i\right)}$) represented in Table 5 of the i number of sub-slip-systems as:

(A5) $$q_{1}^{\left(m\right)}=\frac{1}{X}\sum_{i=1}^{3}h_{i}{\bar{C}}^{\left(i\right)}b_{i}^{2}q_{1}^{\left(i\right)},\;q_{2}^{\left(m\right)}=\frac{1}{X}\sum_{i=1}^{3}h_{i}{\bar{C}}^{\left(i\right)}b_{i}^{2}\;q_{2}^{\left(i\right)}\;\mathrm{and}\;\sum_{i=1}^{3}h_{i}=1$$

where the parameter$\;X=\;\sum_{i=1}^{3}h_{i}{\bar{C}}^{\left(i\right)}b_{i}^{2}=\;{\bar{b^{2}C_{hk.0}}}^{\left(m\right)}$.
